# The impact of free trial acceptance on demand for alternative nicotine products: evidence from experimental auctions

**DOI:** 10.1186/s12954-015-0052-3

**Published:** 2015-06-11

**Authors:** Matthew C. Rousu, Richard J. O’Connor, Maansi Bansal-Travers, James M. Pitcavage, James F. Thrasher

**Affiliations:** Susquehanna University, 514 University Ave, Selinsgrove, PA 17870 USA; Roswell Park Cancer Institute, Elm and Carlton Streets, Buffalo, NY 14263 USA; Geisinger Health System, Danville, PA 17822 USA; University of South Carolina, 915 Greene Street, Columbia, SC 29208 USA

**Keywords:** Tobacco, Smokeless, Experimental auction, Auction, Demand, Smokeless tobacco, Trials, Product trials

## Abstract

**Objectives:**

This study explored the relationship between product trials and consumer demand for alternative nicotine products (ANP).

**Methods:**

An experimental auction was conducted with 258 adult smokers, wherein participants were randomly assigned to one of four experimental conditions. The participants received the opportunity to try, but did not have to accept, one of three relatively novel ST products (i.e., snus, dissolvable tobacco, or medicinal nicotine), or they were placed into a control group (i.e., no trial). All the participants then bid on all three of these products, as well as on cigarettes. We assessed interest in using ANP based on both trial of the product and bids placed for the products in the experimental auction.

**Results:**

Fewer smokers were willing to try snus (44 %) than dissolvable tobacco (64 %) or medicine nicotine (68 %). For snus, we find modest evidence suggesting that willingness to try is associated with greater demand for the product. For dissolvable tobacco or medicinal nicotine, we find no evidence that those who accept the product trial have higher demand for the product.

**Conclusions:**

Free trials of a novel ANP were not strongly associated with product demand, as assessed by willingness to pay. Given the debate over the potential for ANP to reduce the harm from smoking, these results are important in understanding the impact of free trial offers on adoption of ST product as a strategy to reduce harm from tobacco use.

## Introduction

Since the 1990s, tobacco products modified to potentially reduce health risks have been marketed to smokers. One class of such products is a form of smokeless tobacco (ST) modeled on Swedish snus, which is processed to reduce tobacco-specific nitrosamines and other toxicants [1] and has been linked to reduced smoking rates and cancer mortality [2–4]. Snus products were first introduced in the US market by Swedish Match (Revel and General Snus), and later by RJ Reynolds (Camel Snus), UST (Skoal Snus), and Philip Morris (Taboka, Marlboro Snus). Adoption of snus by US smokers has been low [5] despite the fact that overall ST product use appears to be increasing [6].

Other novel ST products have also been introduced into the US market. Lozenge-like products made with powdered tobacco have also been marketed to smokers, including Ariva and Stonewall (Star Scientific). The market in such “dissolvable” tobacco products was later expanded by RJ Reynolds, which introduced Camel Orbs, Sticks, and Strips, and Philip Morris, which test-marketed Marlboro and Skoal Sticks. The FDA’s Tobacco Products Scientific Advisory Committee (TPSAC) reviewed the health impact of dissolvable products, finding that while exclusive use of dissolvable tobacco products was likely less hazardous than cigarette smoking, uncertainties on patterns of use and marketing prohibited firm conclusions on overall public health impact [7]. Despite survey evidence suggesting significant demand among smokers for products promoted as less harmful, available data suggest few smokers have adopted these products. Males appeared to be more likely to have tried dissolvable products in test markets [8]. Perhaps, in response to low demand, most dissolvables had been withdrawn from the market as of 2014.

One way to gauge interest in ST products directly is by offering free trials. This is a common marketing approach designed to reduce innovation resistance by addressing potential cost and risk barriers [9]. Tobacco companies have offered free trials as a way of encouraging ST use [10–12], which is consistent with their past practice of free cigarette giveaways as a promotional strategy [13]. Although the Family Smoking Prevention and Tobacco Control Act of 2009 now prohibits free samples of cigarettes, offering free samples of ST products is allowed.

Experimental economic methods estimate demand by encouraging people to reveal their true valuation of different products ([14, 15]. A key benefit of experimental auctions comes from placing consumers in a real auction, with winners and losers, and where the winners pay for products. This prevents hypothetical bias in surveys and “willingness to pay” studies, where there are no financial consequences to self-reported desires for a product. The experimental auction can be designed so it accurately elicits the demand curve for products [16] and has been employed in recent years to examine issues related to tobacco products. Monchuk et al. [17] examined the value of nicotine in tobacco products using experimental auctions. Thrasher et al. [18, 19] and Rousu and Thrasher [20] used experimental auctions to examine the demand-reducing impact of health warning labels containing prominent pictorial imagery illustrating the consequences of smoking. Rousu et al. [21] used experimental auctions to examine the demand for potentially reduced exposure products (PREPs) relative to conventional cigarettes.

In this paper, we use experimental auctions to explore the link between willingness to accept a free trial of ST and demand for ST. All participants in the auction were smokers but not regular ST users. The participants were offered trials of three alternative nicotine products (ANP): Camel Snus, Ariva, Nicorette, or nothing (in the control group).^1^^1^ These products were selected because they are marketed to smokers (as opposed to ST users) and had been previously studied among smokers [22–24]. All participants then bid on cigarettes and these ANP in an experimental auction, where the bid represents a participant’s demand for one package of the item being bid upon. Results will provide insight into how well product trials translate into demand for ANP. Results will also provide evidence whether participants are equally likely to accept a trial of any ANP. These results are important for public health officials who want to understand whether product trials increase demand for ANP.

## Methods

### Participant recruitment and sample size

The study protocol was approved by the IRBs at the University of South Carolina, Roswell Park Cancer Institute, and Geisinger Medical Center. Participants were recruited by radio ads, newspaper ads, and flyers in Buffalo NY, Columbia SC, and Selinsgrove PA. These sites were chosen because they differ substantially in prevalence of ST use, cigarette taxes, and clean indoor air policies. Eligible study participants were 18 years old and older, currently smoked, were not currently using nicotine replacement or ST products, and had no major medical issues that would warrant exclusion. We excluded current ST users, as we were primarily interested in the interest of current smokers in potential alternatives to cigarettes. So, those with current or regular ST use would likely have different (preformed) opinions about the products relative to more naïve smokers.

Participants were paid US$50 for their participation in the 1-h sessions. Auctions were conducted with eight to sixteen participants at a time with a total of 258 smokers participating between November 2010 and November 2011. This analysis uses a subset of the data reported in Rousu et al. [25], which focused on the impact of information on ANP choices more than the impact of product trials.

### The products

Participants placed separate auction bids for four separate products. Participants bid on three cigarette alternatives: Camel Snus, Ariva Dissolvable Tobacco, and Nicorette Mini-lozenge. These products were chosen to cover three distinct product styles (pouched ST, dissolvable tobacco, and medicinal nicotine, respectively) available in the open market. Participants also bid on Marlboro brand cigarettes, either the red, menthol, gold (light), or menthol gold (light) variety, depending on their individual preference. The fact that participants were able to bid on the most popular brand family in one of four product classes allows comparisons of bids on ST products to bids for cigarettes.^2^^2^

### Experimental conditions

We sought to assess demand for the three ANP, relative to cigarettes, under alternative treatments. Treatment assignment was at the group level—all participants at a given auction session received the same treatment to facilitate the auction protocol. Table 1 shows the treatments and the distribution of participants per treatment in each site. Participants (except for those in the control group) were offered the opportunity to try either Camel Snus, Ariva, or Nicorette, depending on assigned condition (see Table 1). We asked the participants if they would like to try the product but did not require trial to continue in the study. Participants who tried the product were instructed to place the product in their mouth (with supplemental instructions for snus to place between upper lip and gum), to not chew the product, and the leave it in the mouth for up to 5 min. We asked the participants who tried the products to keep their reactions to themselves until after the experiment ended.

**Table 1:** Demographic characteristics–overall and by treatment^a^

	Overall (*N* = 258)	Control (*N* = 62)	Snus trial (*N* = 64)	Nicorette trial (*N* = 67)	Ariva trial (*N* = 65)	Offered and tried the ST product (*N* = 115)	Offered but did not try the ST product (*N* = 81)
Race–white	65 %	66 %	67 %	66 %	60 %	68 %	59 %
Race–black	28 %	31 %	23 %	27 %	32 %	27 %	28 %
Race–other	7 %	3 %	9 %	7 %	8 %	5 %	12 %
Age–under 30 years^b^	38 %	27 %	36 %	39 %	51 %	44 %	38 %
Age–30 to 50 years	38 %	35 %	41 %	36 %	38 %	39 %	37 %
Age–over 50 years^b^	24 %	37 %	23 %	25 %	11 %	17 %	25 %
Female	44 %	39 %	41 %	51 %	45 %	38 %^c^	55 %
Income–below 30 K	57 %	56 %	63 %	49 %	58 %	54 %	60 %
Income–between 30 and 60 K	10 %	19 %	09 %	10 %	11 %	14 %	14 %
Income–over 60 K	15 %	10 %	11 %	13 %	17 %	10 %	10 %
Income–chose not to reveal	18 %	15 %	17 %	27 %	14 %	22 %	16 %
Moderately or very worried about future quality of life	61 %	61 %	59 %	64 %	58 %	62 %	59 %
Prior smokeless tobacco use	43 %	39 %	50 %	43 %	38 %	55 %^c^	28 %
Observations–NY	36 %	39 %	33 %	36 %	38 %	38 %	30 %
Observations–SC	34 %	35 %	36 %	37 %	29 %	32 %	37 %
Observations–PA	29 %	26 %	31 %	27 %	32 %	30 %	31 %

^a^Auctions conducted from November 2010–November 2011

^b^The only demographic characteristics that differed across treatment groups at a significance level of 0.05 or less was the over 50 age group (*p* < 0.01). No other differences in demographic characteristics were found to be significant at the 5 % level using a chi-squared test

^c^The only demographic or background characteristics where there were statistically significant differences in the percentage who tried ST products was for females (*p* < 0.05) and for those who had used smokeless tobacco at some point in the past (*p* < 0.01)

For analytic purposes, we distinguished those who declined the trial offer from those who accepted it (since the non-triers would bias the effect toward the null). While participants placed a separate bid on each of the four products, only one product was chosen as the binding product (i.e., the product that would actually be auctioned). This product was selected after all bidding by a random draw to ensure participants would not decrease their bids due to thinking they might win more than one tobacco product [26].

### Experimental design

Data were collected using the random n^th^ price auction mechanism (Shogren et al. [27], in which participants are initially given enough money to compensate for their time and to provide them with more than enough money to pay the “winning” price for the product of interest.^3^^3^ Participants are told that this auction is different from other auctions in that they can only bid once (on a product), and it is in their best interest to submit a bid equal to the full price they would pay for the product. Once all bids are collected, bids are sorted from highest to lowest. After eliminating the highest bid, one bid is then selected randomly. The (n-1) participants that bid more than this randomly selected price purchase the product, *paying the price of the selected n*^*th*^*highest bid*; the participants who bid less than (and the participant whose bid is equal to) the *n*^*th*^ highest bid do not purchase the product. In this mechanism, a participant will not pay more than their submitted bid for the product. This auction is “demand revealing” in that it is in a participant’s best interest to bid his or her true value (demand) for the product because the amount the auction winners pay is determined by another subject’s bid, not their bid. Someone who bids higher than her true value for the product could end up paying more than that true value, while someone who bids lower than her true value may miss out on a profitable purchase if the randomly selected binding price is less than her true value but higher than the bid she submitted.

### Procedures

After participants arrived and signed a consent form, they filled out a brief survey on their tobacco use history (Step 1). Next (Step 2), participants received a detailed explanation of the auction mechanism (both orally and in writing), emphasizing that it was in their best interest to bid their true value for the products. Participants also took a quiz to ensure they understood the auction procedures. The monitor went through the answers to the quiz questions with participants. Next, the participants participated in a practice auction for two candy bars (Step 3) that demonstrated the real procedures by having participants place bids for different candy bars in different rounds, including random selection of the binding product. The detailed explanations, the quiz, and the practice auction helped ensure that all participants understood the procedures. In Step 4, participants received their randomly assigned treatment (trial of Camel Snus, Ariva, Nicorette, or no trial). Then (Step 5), the participants placed separate, private bids on each of the four products. As it was impractical to randomize the order of bidding within and across treatments, participants in all treatments always placed their first bid on the Snus, their second bid on the Ariva, their third bid on the Nicorette, and their final bid on the cigarettes. After all four bids were submitted, a random draw was conducted to determine which product was the binding product, followed by a random draw to determine the n^th^ price (Step 6). This determined who won products, which product, and how much the winners would pay. Finally (Step 7), participants filled out a post-auction questionnaire, winners exchanged money for their product, and the experiment ended. The post-auction questionnaire included an item that asked “How much do you think the Camel Snus you bid on in the auction would cost in a local store?”. There were three similar questions for cigarettes, Ariva, and Nicorette.

### Analysis

To examine the impact of demographic- and tobacco-related characteristics on product trial, along with the product being offered to participants, we used probit models. If participant *i* tried the product, we define this as *D*_*i*_ = 1. To examine the impact, the product and demographic and tobacco-related characteristics have on the probability of a accepting a product trial, a probit model is used and is shown in the following equation:

1 $$ \mathrm{Prob}\left({D}_i=1\right)=f\left(\alpha +\delta \hbox{'}{L}_i+\beta {X}_i+\gamma \hbox{'}{C}_i+{\varepsilon}_i\right) $$

Where α is an intercept term; *L*_*i*_ is a dummy variable that represents the ST product a person was offered and δ’ is the associated coefficient; *X*_*i*_ is a vector that represents the demographic characteristics of participant *i* and *β’* is the associated coefficient vector; *C*_*i*_ is a vector that represents the tobacco-related characteristics of participant *i* and γ’ is the associated coefficient vector, and ε_*i*_ is the error term. To examine the possible impact of product trials and participant characteristics on demand for ST, we estimated a random effects regression model with the following equation:

2 $$ BIDit={\upalpha}_i+\upmu '{\mathrm{P}}_i+\beta '{X}_i+\updelta '{\mathrm{L}}_i+\pi '{T}_i+\upgamma '{\mathrm{C}}_i+{\boldsymbol{\upvarepsilon}}_{it}. $$

In equation 2, *BIDit* is participant *i*’s bid for the ANP *t* (where *t* = Snus, Ariva, or Nicorette), α_i_ is a random effects intercept term; *P*_*i*_ is a dummy variable that represents the product a person was bidding on and μ’ is the associated coefficient; *L*_*i*_ is a dummy variable that represents the ST product a person was offered to try and δ’ is the associated coefficient; *X*_*i*_ is a vector that represents the demographic characteristics of participant *i* and *β’* is the associated coefficient vector; *T*_*i*_ is a vector that represents whether participant *i* accepted or rejected an opportunity to try an ANP and *π’* is the associated coefficient vector; C_*i*_ is a vector that represents the demographic and tobacco-related characteristics of participant *i* and γ’ is the associated coefficient vector; and ε_*it*_ is the error term.

## Results

Table 1 presents the background and demographic characteristics of our sample. We had 258 participants across the control group and three treatment groups. Sixty-five percent of our sample identified themselves as white, with 35 % identifying as non-white. Our sample was split somewhat evenly between those under 30 years old, between 30 and 50 years old, and older than 50 years old. For those who were over 50 years old, we found a statistically significant difference in the percentages across treatments using a chi-squared test (*p* < 0.01). For all other demographic characteristics, we found no statistically significant differences in demographic or background characteristics across any of the treatments. Approximately two-thirds of the participants in the Ariva (68 %) and Nicorette (64 %) treatments tried the products, while fewer participants in the Camel Snus group (44 %) tried snus. The differences between the percentages of people who tried snus relative to Ariva or Nicorette were statistically significant at the 1 % level using a *t* test. Table 1 also shows how the characteristics differed among those who accepted the offer to try the product and those who were offered a free trial but rejected it. Females were less likely to accept the free trial of the ANP while those who had tried ST in the past were more likely to accept the free trial of the ANP.^4^^4^

Results from the probit model are shown in Table 2. Those who were offered Nicorette and Ariva were more likely to try an ANP relative to those offered snus. Those who considered themselves neither white nor black (i.e., in the other race category) were less likely to try an ST product than whites. Those who had previously tried ST products were more likely to accept the free product trial.

**Table 2:** Probit model examining the probability that a participant in one of the three trial groups tried a product (*N* = 196)

Characteristics		Coefficient (standard error in parentheses)
Product offered to participant in trial	*Intercept*	−0.70
		(0.57)
	*Nicorette*	0.51^b^
		(0.25)
	*Ariva*	0.72^b^
		(0.25)
Age		0.00
		(0.01)
Income		0.11
		(0.09)
Education		0.03
		(0.10)
Female		−0.19
		(0.23)
Race/Ethnicity	*Black* vs. *White*	−0.09
		(0.29)
	*Other* vs. *white*	−0.80^a^
		(0.39)
State	*South Carolina (*vs. *New York)*	−0.40
		(0.27)
	*Pennsylvania (*vs. *New York)*	−0.21
		(0.28)
Ever used smokeless tobacco		0.80^b^
		(0.24)
Worried about their quality of life because of smoking		0.10
		(0.22)
Estimate of the cost of smokeless tobacco products		−0.00
		(0.01)

^a^Statistically significant at the 0.05 level

^b^Statistically significant at the 0.01 level

The mean bids from the auctions are presented in Table 3. The bid for snus was lower than the bid for Ariva, which was lower than the bid for Nicorette, which was lower than the bid for cigarettes. However, using Bonferroni adjusted *p* values, differences between snus and Nicorette are statistically significant (*p* < 0.01), and the differences between cigarettes and all ANP are statistically significant (*p* < 0.01), but differences between Ariva and snus and Ariva and Nicorette are not statistically significant at conventional levels (*p* = 0.129 and 0.315). The bid for snus among participants in the group that was offered Nicorette was higher (*p* < 0.05) than bids for snus in other treatments. There were no other statistically significant differences in the unconditional results based on which treatment participants were placed.

**Table 3:** Mean bids across treatment groups

	Overall^a^ (*N* = 258)	Control (*N* = 62)	Snus trial (*N* = 64)	Nicorette trial (*N* = 67)	Ariva trial (*N* = 65)
Bid Snus	US$1.00	US$0.72	US$0.60	US$1.55^b^	US$1.13
	(1.86)	(1.12)	(0.98)	(1.53)	(1.90)
Bid Nicorette	US$1.87	US$2.04	US$1.54	US$1.87	US$2.03
	(2.62)	(2.23)	(2.23)	(3.13)	(2.78)
Bid Ariva	US$1.40	US$1.44	US$0.91	US$1.78	US$1.47
	(1.92)	(1.42)	(1.42)	(2.11)	(2.42)
Bid Cigarettes	US$4.06	US$4.09	US$3.30	US$4.16	US$4.76
	(2.34)	(1.84)	(1.89)	(4.09)	(2.44)

(Standard deviations in parentheses)

Difference in bids for those who tried snus vs. those who chose not to try snus is statistically significant at the 5 % level

^a^Differences in bids across all four products are statistically significant at the 5 % level using a 2-sided *t* test

^b^Differences in bids for snus in the group that received the snus trial and those that received the Nicorette trial are statistically significant at the 5 % level using a 2-sided *t* test

To further examine the interaction between product trials and product demand, we examine the bids for the ST products for those who accepted and those who refused the product trial. These results are shown in Table 4. The difference in bids for snus is lower for those who accepted the trial at the 5 % level using a 2-sided *t* test.

**Table 4:** Differences in bids for those who tried and did not try the ST products

	Accepted snus trial: bid for snus	Rejected snus trial: bid for snus	Accepted Nicorette trial: bid for Nicorette	Rejected Nicorette trial: bid for Nicorette	Accepted ariva trial: bid for ariva	Rejected ariva trial: bid for ariva
N	28	36	43	24	44	21
Bid	0.93^a^	0.34	1.43	2.66	1.58	1.26
	(1.10)	(0.80)	(1.43)	(4.60)	(2.44)	(2.42)

(Standard deviations in parentheses)

^a^Difference in bids for those who tried snus vs. those who chose not to try snus is statistically significant at the 5 % level

Results for the random effects regression are shown in Table 5. Participants bid more for Ariva and Nicorette than for snus. Those who were offered and rejected Nicorette bid more for the ST products than others, and the results were statistically significant in two of the three models. We found no other impact of accepting or rejecting a product trial on bid prices. Those who had a higher estimate of the store price for ANP placed higher bids for the ANP in the auction. The effect was statistically significant (*p* < 0.05) but small. For every US$1 increase in estimated store prices, participants bid US$0.02 more for the ST product. We also find that participants who bid higher for cigarettes also bid higher for ST products. For every US$1 increase in a bid for cigarettes, participants bid US$0.40 more for ST products.

**Table 5:** Random effects regression-dependent variable is bid on ST product (*N* = 258)

Characteristics		Model 1	Model 2	Model 3
Product bid upon	*Intercept*	0.97	−1.60	−1.20
		(0.71)^+^	(0.72)	(0.72)
	*Nicorette*	0.89***	0.89***	0.89***
		(0.13)	(0.13)	(0.13)
	*Ariva*	0.35***	0.35***	0.37***
		(0.13)	(0.13)	(0.13)
In treatment where offered, but rejected opportunity to take a trial, relative to control group	Offered but rejected Snus	−0.54	−0.06	−0.13
		(0.39)	(0.35)	(0.35)
	Offered but rejected Nicorette	0.95**	0.87**	0.50
		(0.46)	(0.41)	(0.40)
	Offered but rejected Ariva	−0.08	0.05	0.01
		(0.46)	(0.41)	(0.40)
In treatment where offered, and accepted opportunity to take a trial, relative to control group	Tried Snus	−0.07	0.11	0.08
		(0.42)	(0.38)	(0.37)
	Tried Nicorette	0.20	0.27	0.23
		(0.38)	(0.34)	(0.33)
	Tried Ariva	0.18	−0.04	−0.06
		(0.38)	(0.34)	(0.33)
Age	−0.00	0.01	0.00	
	(0.01)	(0.01)	(0.01)	
Income	−0.09	−0.08	−0.06	
	(0.10)	(0.09)	(0.09)	
Education	0.12	−0.08	0.15	
	(0.11)	(0.09)	(0.10)	
Female	0.15	0.14	0.11	
	(0.28)	(0.25)	(0.24)	
Race/Ethnicity	*Black* vs. *white*	0.16	0.03	−0.01
		(0.32)	(0.28)	(0.29)
	*Other* vs. *white*	0.57	0.05	0.13
		(0.48)	(0.43)	(0.42)
State	*South Carolina (*vs. *New York)*	−0.82	0.09	0.03
		(0.31)	(0.29)	(0.30)
	*Pennsylvania (*vs. *New York)*	−1.04***	−0.87***	−0.84***
		(0.32)	(0.29)	(0.28)
Ever used smokeless tobacco		0.10	0.09	0.04
		(0.29)	(0.26)	(0.25)
Worried about their quality of life because of smoking		−0.01	0.06	−0.01
		(0.24)	(0.22)	(0.21)
Estimate of the cost of smokeless tobacco products		0.02**	0.02***	0.02***
		(0.01)	(0.01)	(0.01)
Bid for cigarettes			0.40***	0.38***
			(0.05)	(0.05)
Participant preferred menthol cigarettes				−0.06
				(0.23)

**Statistically significant at the 0.05 level

***Statistically significant at the 0.01 level

+Robust standard errors are reported in parentheses

For a sensitivity analysis, we examined an alternative view of demand—whether the participant placed a non-zero bid for the ANP. The results were similar: for snus, those who tried snus were more likely to place a positive bid. However, we found no evidence that product trials of Ariva or Nicorette were more likely to bid a positive amount for those products. Those results are available from the authors upon request.

## Discussion and conclusion

While product trials (e.g., free samples) have been utilized by the tobacco industry as a potential way to increase demand, we found evidence that trial mattered for snus, but not other ANP: smokers who were willing to try snus had a higher demand for snus, as assessed by their bids. Indeed, while a majority of participants declined to try snus, the minority who tried it exhibited greater demand, suggesting that experience of use may be influential, that factors related to snus trial (e.g., comfort with placing tobacco vs. a more medicinal appearing product in the mouth), or unmeasured differences between those who tried vs. did not try snus, were likely to cause higher demand. For other products, the willingness to sample the product was not correlated with demand.

We found modest evidence that those who were offered but rejected the opportunity to try Nicorette bid more for ST products. This provides some evidence that there is a subset of smokers who may view Nicorette as substantially different from other products that were auctioned, perhaps reflecting a greater familiarity with Nicorette, due to its having been on the market (as a cessation aid) for a longer period of time. Those who perceived a higher retail price bid more for the products, suggesting that knowledge of outside prices influence bids.

Further work is needed to understand the factors that influence uptake and continued use of potentially reduced risk tobacco products. Some evidence suggests that e-cigarettes, for example, are more appealing to smokers than ST or nicotine replacement products [28]. Indeed, e-cigarettes appear to approximate the sensory experience and bolus of nicotine that comes with smoking conventional cigarettes compared to consuming the ANP examined in this study. Hence, trial of e-cigarettes may be more likely to influence demand. Given the rapid increase in e-cigarette use [29] future research should examine whether product trials for e-cigarettes augment demand.

This study is subject to some limitations. First, because participants were not required to try the products, we cannot rule out self-selection bias in the impact of free trials on demand. That said, we only found evidence that males (vs. females) and those who previously tried ST products were more likely to accept the free trial. Second, we did not have sufficient sample size to randomize the presentation order, and we appear to see an impact based on ordering. That said, many studies have found differences in bids across alternative products or labels regardless of ordering. Third, participants were assigned to try only one of the three products offered, so we cannot directly compare the impact of trials on demand within subjects across products. Furthermore, experimental auctions in a controlled setting may not adequately represent what happens in the real world. However, there are some consistency checks that make us believe that our experimental auction captured what would occur in the market. Our finding of relatively lower demand for ANP vs. conventional cigarettes is consistent with the relatively low demand found for these products on the market [5, 10, 25, 28].

Those who accept a trial of ANP are showing that they view the product as worth trying for free. In this regard, it seems logical to expect that free trials would be correlated with higher demand, and indeed, might lead to higher demand for ANP. However, we do not find widespread evidence of this. We only find that the willingness to try snus is positively correlated with demand. For Nicorette and Ariva, free trial did not appear to increase demand. Given the debate over the potential for ANP to reduce the harm from smoking, these results are important in understanding the impact of free trial offers on adoption of ANP as a strategy to reduce harm from tobacco use.

## Footnotes

1 Nicorette is not a smokeless tobacco product, but rather an FDA-approved smoking cessation medication. For ease of exposition, however, when we discuss “ST products”, we are referring collectively to Ariva and Snus, and when we discuss “alternative nicotine products (ANP)”, we are referring to all three products.

2 Ideally, we could have presented each smoker with an opportunity to bid on his or her favorite brand. However, logistically (and for budgetary reasons) that would have been difficult, as we would have had to have hundreds of different packs of cigarettes ready to sell in the auction to be ready for all possible combinations of participant cigarette preferences.

3 This differs from a closed tender because there is no negotiation. Further, a standard definition of an auction involves collecting bids from participants and selling the product or products to the highest bidder or bidders.

4 The order of product presentation across sessions was fixed (i.e., not randomized) because we did not have enough sessions to properly randomize the presentation order. We first had participants bid on snus, then Ariva, then Nicorette, and their last bid was for cigarettes. The mean bids, coincidentally, increased on each subsequent product. That said, bids change because of product attributes, not rounds. While some ordering effects could occur (particularly because of framing), studies that have varied the products across rounds have found that the product characteristics tend to be the primary cause of differences in bids. For example, Huffman et al. [30] found that bids for GM-labeled food products were lower than plain-labeled food products, regardless of whether the first set of bids were on the plain-labeled or GM-labeled foods. Another example is Thrasher et al. [19] which did find a small difference based on ordering, but found that bids for cigarettes with graphic pictorial labels were lower than cigarettes with only a text warning, regardless of the ordering.
